# Enhanced recovery after surgery: comes out to the Sun

**DOI:** 10.1186/s12871-023-02236-4

**Published:** 2023-08-14

**Authors:** Mohamed R. El Tahan, Akhilesh Pahade, Manuel Ángel Gómez-Ríos

**Affiliations:** 1 Cardiothoracic Anaesthesia Unit, Department of Anaesthesia, Intensive Care, and Pain Management, College of Medicine, Mansoura University, Mansoura, Egypt; 2 Anesthesiology Department, College of Medicine, Imam Abdulrahman Bin Faisal University, Dammam, Saudi Arabia; 3 SRMS Institute of Medical Sciences, Bareilly, India; 4 Anesthesiology and Perioperative Medicine, Complejo Hospitalario Universitario de A Coruña, A Coruña, Galicia, Spain; 5 Departmento de Anestesiología, Complejo Hospitalario Universitario de A Coruña, Xubias de Arriba, 84, A Coruña, 15006 Spain

**Keywords:** Enhanced recovery after surgery, Perioperative medicine, History, Protocols, Outcomes, Evidence-based, Meta-analysis, Research

## Abstract

ERAS programs aim to reduce the length of hospital stays and lower costs, and minimize the risk of postoperative complications and readmissions while enhancing the overall patient experience. BMC Anesthesiology has initiated a new collection on ERAS, urging investigators to conduct large-scale, high-quality studies that address the existing knowledge gap.

The safety of perioperative patients has garnered significant attention from various global scientific communities intending to minimize patient harm. The Anesthesia Patient Safety Foundation (APSF) has identified ten priorities and corresponding measures to enhance patient safety. These initiatives emphasize the significance of reinforcing teamwork and fostering multidisciplinary collaboration [1]. This highlights the essential expansion of roles and the evolving landscape of anesthesiologists, the primary physicians involved in perioperative care. They play a vital role in enhancing patient safety by developing and evaluating the feasibility and effectiveness of various safety measures, optimizing resource utilization, and improving financial aspects, especially in delivering high-quality perioperative care to patients with complex medical conditions and multiple risk factors. Perioperative medicine strives to decrease perioperative complications and adverse effects, as up to 50% of these incidents can be preventable by modulating the neuroendocrine-metabolic and inflammatory-immune responses to anesthesia and surgery [2]. This includes measures to prevent and manage perioperative infections and airway, pulmonary, cardiovascular, and renal complications. It also involves maintaining established safety standards and utilizing checklists [3]. These efforts can result in a reduced requirement for intensive care unit admissions, shorter hospital stays, decreased hospital readmissions, lower mortality rates, and overall healthcare cost reduction. Consequently, there is an increasing need to enhance the existing training programs in anesthesiology [4].

Enhanced Recovery After Surgery (ERAS) protocols are multidisciplinary recommendations for perioperative care aimed at improving recovery through research, education, audit, and implementing of evidence-based practices. Ken Fearon and Olle Ljungqvist initiated these protocols as a collaborative effort in 2001, leading to the development of the first evidence-based consensus ERAS protocol for patients undergoing colonic surgery in 2005. As the body of evidence supporting the feasibility and effectiveness of ERAS grew, the ERAS Society was established in Amsterdam in 2010. Since then, the society has developed twenty-two consensus guidelines and protocols on ERAS in various specialties as of May 20, 2023. These specialties include anesthesia, bariatric surgery, breast surgery, cardiac surgery, colorectal surgery, cytoreductive surgery, emergency laparotomy, gastrectomy, gastrointestinal surgery, gynecology, head and neck surgery, liver surgery, liver transplantation, elective abdominal and pelvic surgery in low-middle-income countries (LMICs), lumbar spinal fusion, neonatal surgery, obstetrics, esophagectomy, orthopedic surgery, pancreatic surgery, thoracic surgery, urology, and vascular surgery.

ERAS guidelines aim to reduce the length of hospital stays and lower costs, and minimize the risk of postoperative complications and readmissions while enhancing the overall patient experience. These goals align with the objectives of perioperative medicine, which encompasses a holistic approach to patient care during the perioperative period.

Based on scientific knowledge, there are 24 crucial components of ERAS care, and it is theorized that no single element alone can improve surgical outcomes [5]. Since multiple disciplines contribute to the implementation of ERAS protocols, it is not only important to adopt a multidisciplinary approach but also to ensure compliance from all contributing specialties to achieve optimal results [5]. It is worth noting that achieving a compliance rate of 70% or higher with each component of the ERAS protocols can reduce the occurrence of significant postoperative complications and shorten hospital stays. Despite the evidence supporting improved postoperative outcomes and recovery, the implementation of ERAS practices has been slow and inconsistent across different hospitals, possibly due to the involvement of multiple disciplines. Anesthesiologists and perioperative physicians play a critical role in successfully implementing ERAS protocols and should take the lead in ensuring seamless coordination of all components. This encompasses various aspects such as patient counseling/education, prehabilitation, and optimization, minimizing fasting periods, utilizing multimodal analgesia with careful opioid management, promoting early mobilization, and facilitating a swift return to a normal diet and lifestyle. To increase compliance rates, it is necessary to implement approved institutional protocols and provide education and training.

Several meta-analyses have been conducted to assess the effectiveness of different ERAS protocols in achieving the objectives of ERAS. An umbrella review of these meta-analyses encompassed 23 studies, comprising both interventional and observational ERAS studies. The findings revealed a reduction in hospital stay (mean difference: -2.349 days; 95% confidence interval: -2.740 to -1.958) and costs (mean difference: -$639.064; 95% confidence interval: -933.850 to -344.278) without an increase in mortality across all surgical procedures involving ERAS patients. Furthermore, in orthopedic surgery, ERAS was associated with a decrease in the 30-day mortality rate (odds ratio: 0.40; 95% confidence interval: 0.23 to 0.67). However, it was also found to increase morbidity following laparoscopic gastric cancer surgery (risk ratio: 1.49; 95% confidence interval: 1.04 to 2.13) and the readmission rate following open gastric cancer surgery (risk ratio: 1.92; 95% confidence interval: 1.00 to 3.67) [6].

A meta-analysis of 27 studies involving 6345 gynecologic oncology patients demonstrated that implementing ERAS reduced hospital stays by 1.6 days, a 32% decrease in complications, a 20% reduction in readmissions, and a mean cost savings of $2129 [7]. Noba et al. [8] reported a decrease in hospital stays by 2.22 days and a lower incidence of complications (risk ratio: 0.71; 95% confidence interval: 0.65–0.77; p < 0.00001) in six randomized controlled trials (RCTs) involving 1777 patients in the ERAS group and 1,962 patients in the standard care group following hepatic surgery. These findings also reduced healthcare costs (standardized mean difference: -0.98; 95% confidence interval: -1.37 to -0.58; p < 0.0001). Similar results have been reported in patients undergoing spine, pediatric, bariatric, thoracic, and prostate surgery with the implementation of ERAS [9–13].

Similar advantages have been observed in the context of emergency surgery. Hajibandeh et al. [14] conducted a meta-analysis involving 1334 patients from six comparative studies who underwent emergency abdominal surgery and found that the implementation of ERAS protocols led to expedited bowel recovery, shorter hospital stays, and reduced risks of major complications, pulmonary complications, and surgical site infections. Notably, these benefits were achieved without significant differences in mortality or readmission rates.

The majority of ERAS trials have predominantly focused on adult patients undergoing elective surgery. However, the potential benefits of ERAS in geriatric, pediatric, and emergent surgery populations have been less explored. Consequently, further investigation is warranted to determine whether the advantages of ERAS can be extended to these specific cohorts as well [15]. Furthermore, additional research is needed to identify barriers and evaluate the effectiveness of proposed solutions to implement institutional ERAS protocols, focusing on enhancing educational and training programs and activities specific to ERAS.

In the future, we hope to better understand risk stratification tools that can help identify patients who are most likely to benefit from ERAS protocols. Additionally, efforts should be made to optimize perioperative opioid and fluid utilization. Undoubtedly, ERAS protocols will continue to be embraced by more institutions, perioperative physicians, and surgeons in the coming days. However, it is important to remember that starting a job is often easy, but maintaining and successfully concluding it can be extremely challenging. Therefore, it is crucial to devise alternative and backup plans and implement constant quality checks and controls to address setbacks logically. Further research will hopefully address the existing knowledge gaps in the coming years.

## Data Availability

Not applicable.
